# Teacher Mobility: What Is It, How Is It Measured and What Factors Determine It? A Scoping Review

**DOI:** 10.3390/ijerph19042313

**Published:** 2022-02-17

**Authors:** Claudia Palma-Vasquez, Diego Carrasco, Mónica Tapia-Ladino

**Affiliations:** 1 Faculty of Education, Universidad Católica de la Santísima Concepción, Concepción 4090541, Chile; mtapia@ucsc.cl; 2 Center for Research in Occupational Health (CiSAL), Department of Experimental and Health Sciences, Pompeu Fabra University, 08003 Barcelona, Spain; 3 Centro de Medición MIDE UC, Pontificia Universidad Católica de Chile, Santiago 7820436, Chile; dacarras@uc.cl

**Keywords:** teacher mobility, teacher turnover, teacher attrition, teacher workforce, teacher retention, scoping review

## Abstract

Teacher mobility represents a serious problem due to the instability of the teaching force that has persisted over time in many countries. Therefore, retaining qualified teachers represents a challenge given the difficulty of having the necessary workforce to face the educational challenges of each year. Our objective was trying to identify how mobility is understood and measured, that is, teacher turnover and attrition, and to identify the results of the related factors according to the different perspectives. The PRISMA-Scr protocol was used, which establishes the information that should be included in a systematic review. The following key phrases were used: “teacher rotation” or “teacher mobility” or “teacher desertion” or teacher leavers or teacher stayers. The databases used were Web of Science, Scielo Citation Index and Google Scholar, which yielded an initial total of 760 documents published between 2008 and 2018, that after identification, screening, eligibility, and inclusion processes, were reduced to 213. The selection of articles was carried out independently by two researchers using a structured and recursive hierarchical strategy. The existence of multiple ways of defining and measuring teacher mobility was identified and a definition based on two perspectives was proposed that summarizes the conceptual and operational findings, which are indirect and direct mobility. The first refers to the intention to leave and the second to leave. We have identified more evidence related to direct studies of a quantitative approach and focused on teachers with medium or short experience. The factors associated with mobility were identified based on the approaches used and a key element was identified when distinguishing teacher mobility, which is voluntary and involuntary mobility. We identified multiple factors associated with teacher mobility, among which the precarious working environment, poor organizational conditions such as lack of leadership and support among colleagues, excessive workload and low self-efficacy stand out. The limitations of this study are discussed. The findings of this study are highly relevant since they allow proposing medium or short-term policies, such as improving the organizational conditions of the school to promote the retention of the teaching workforce.

## 1. Introduction

Education is fundamental for the development of societies, and in every educational process, teachers play a crucial role in student achievement and learning [1,2]. In this scenario, retaining qualified teachers is a central issue, especially considering the high rate of teachers leaving schools or the teaching career around the world [3,4,5,6,7].

Teacher retention is a challenge to guarantee an adequate number of qualified teachers to meet the educational challenges of schools in localities or countries [1,8]. On the other hand, the lack of teachers due to teacher mobility, either by rotation, that is, by changing schools, or by abandonment, that is, the interruption of the teaching occupation in order to dedicate oneself to other work, constitutes a severe problem of a decrease in the teacher workforce [3,9,10].

Teacher turnover and attrition rates differ from country to country; however, evidence suggests that it is a phenomenon that has generally increased over time in all countries [1]. In the United States and England figures reach 50% of lost teachers by the fifth year of their tenure [10]. In Australia, the situation is similar, with a decrease of 40% and 50% of the teaching force with five years of teaching experience [11]. In the Netherlands, the drop-out rate of teachers is estimated to be around 15%; however, this pattern has increased over the last 30 years [12]. Recent studies emphasize that faculty shortages are a high-impact problem that has gone unnoticed and is often more severe than estimated [13]. In fact, the recent report by Ingersoll, Merrill, Stuckey, and Collins [4], about the characteristics of the teaching force in the United States emphasizes that one of the trends that have been maintained over time is the instability of the teaching profession due to high turnover and drop-out rates. In South American countries such as Chile, the situation is equally worrying. A recent report found that between 2005 and 2016, about 20% of teachers left the profession before completing five years of practice, and 70% did not continue teaching in the school where they entered the first year. That study also found that the rate of temporary withdrawal of teachers with less work experience was high, with a loss of between 6% and 12% of teachers each year [6]. Other studies from Chile have found that teachers last about 3.1 years on average on their first job [5]. Teaching spells are shorter among younger teachers, those who work in private subsidized schools, in rural contexts, and shorter among more socioeconomically disadvantaged schools [5]. Other studies assert that teacher turnover patterns in subsequential teaching spells present similar rates. Thus, reaffirming the idea that teaching spells are similar in length, regardless of whether these are the teachers in their first, second, or third job posts. Some variation is observed: teaching turnover after the first job is 2% to 3% higher than their first job and working and school conditions remain relevant [14].

In summary, the literature shows that teacher mobility, whether through rotation or attrition, is a priority issue. To a greater extent, it seems to affect teachers with less experience and those who work in contexts of high vulnerability or greater poverty (e.g., rural areas with high vulnerability indices, which serve students with fewer resources) [13,15,16,17,18,19]. Teacher attrition is of great concern because teachers reach their level of expertise after the first years of their profession [20]. As such, a high percentage of teachers would leave their schools or leave the profession altogether before reaching their peak performance [21]. Therefore, a high proportion of teachers may never achieve the expected level of expertise in schools serving the most disadvantaged students, thus impacting the quality of their teaching, and widening the inequality gap [5,13,22,23].

Regarding the effects of teacher turnover on students learning, it has been found to affect students directly and indirectly. It affects students directly due to the interruption of the teaching and learning process, either by the change of teachers itself or the difficulties of finding replacement teachers. Furthermore, it affects students indirectly because it deteriorates the functioning of the schools as a whole [24]. Therefore, teacher mobility appears to harm the performance of all students in the school [25]. Moreover, since teacher mobility also results in the loss of experienced teachers from schools, it brings an additional cost related to the selection and training of new professionals for educational organizations [26], which may be difficult for poorer schools to afford.

Teacher mobility is relevant for the current educational environment [1] and to the occupational health of teachers who remain and those who leave [27]. For this reason, there is increasing evidence on the factors that may be associated with this phenomenon [3,4,5,10,15,16,28]. In this area, a sufficiently large body of literature allows us to approach the factors associated with this phenomenon [3,10,16,29,30,31,32,33,34], which have approached mobility from two different perspectives. These perspectives refer to the phenomenon before and after it occurs, that is, the intention to leave and leave. However, this is not clearly specified in the specialized literature, even though it is key to understanding teacher mobility. Furthermore, no known scoping or systematic reviews conceptually or operationally define teacher turnover or attrition, nor do they provide up-to-date information on the components most associated with this phenomenon according to different perspectives. The last review related to teacher attrition and retention identified is from 2008 [10]. Therefore, we aim to identify, order, analyze and draw conclusions from recent scientific production on how teacher turnover and attrition are understood and measured. Moreover, we aim to identify which factors are related according to the different perspectives mentioned.

## 2. Materials and Methods

The design of this study is theoretical. Specifically, it is a scoping review [35] because it aims to identify the state of the current literature, its main approaches, and research gaps [36]. To achieve this, we followed the recommendations of the PRISMA Statement [37]. Specifically, we used the PRISMA extension protocol for scoping reviews (PRISMA-Scr) proposed by Tricco et al. [38], which establishes the fundamental elements that should be included in a scoping review. The checklist that contains these items can be found in Table S1 of the Supplementary Materials.

All studies published between 2008 and 2018 were included in the document search process to obtain evidence from the last ten years, after the Borman and Dowling [10] review about teacher mobility and retention published in 2008. This search was conducted using the following key phrases: “teacher mobility” or “teacher turnover” or “teacher attrition” or teacher leavers or teacher stayers, in that order. The databases used were Web of Science, Scielo Citation Index, and Google Scholar for its high reaching power, which yielded an initial total of 760 documents. After the process of identification, screening, eligibility, and inclusion, they were reduced to 213.

### 2.1. Inclusion and Exclusion Criteria

To be included in this review, we applied the following criteria to screen for qualified studies:

(a) The study population was teaching staff, regardless of their level of practice.
(b) The outcome of the studies was mobility in any of its perspectives (before, i.e., turnover or attrition intention or its predictors; or after, i.e., turnover or attrition teacher).
(c) Survey data should have been collected within the last 20 years.
(d) Published in a peer-reviewed journal

The exclusion criteria included:

(a) Papers that were commentary articles, conference proceedings, and professional reports
(b) Grey literature
(c) Research focused on other populations (i.e., students, university professors)

### 2.2. Phase 1: Identification

This first phase included studies published in any language between 2008 and 2018 using the key phrases. The results of the search in the databases: Web of Science (Main Collection), Scielo Citation Index, and Google Scholar yielded a total of 314, 6, and 440 documents, respectively. The details of the strategy used can be found in Table 1.

We used the EndNote X8 Bibliographic Manager to manage the documents and identified 41 duplicate documents.

### 2.3. Phase 2: Screening

The documents that met the minimum quality criteria were selected at this stage. We were interested in selecting evidence that would provide robustness to the study of the factors related to teacher turnover and attrition. Therefore, it was decided to exclude grey literature and those documents that do not require peer or editorial board review to be published. Therefore, 275 master’s and doctoral theses, 18 books or book chapters, 5 conferences, seminar, or workshop proceedings, 13 working papers, and 2 projects were excluded. In addition, seven documents were excluded for various reasons (three were not found, two corresponded to erroneous citations, and two corresponded to works prior to 2008). In this process, a total of 320 documents were discarded.

### 2.4. Phase 3: Eligibility

To ensure the suitability of the 399 documents that remained from the previous stage, two reviewers independently carried out the document selection process (CP and FN), using a structured and recursive hierarchical strategy. First, a rubric was developed to analyze the title and abstract of the documents, according to specific criteria such as type of study, population, outcome, and others. Secondly, a pilot evaluation was carried out to assess the clarity of the rubric during its application. Any doubts between reviewers were clarified to ease the process of review. Thirdly, titles and abstracts were read to extract preliminary information and determine the inclusion criteria for the final stage.

### 2.5. Phase 4: Inclusion

In the inclusion phase, the two reviewers (CP and FN) independently assessed the selected studies through the complete reading of the paper, considering of course the inclusion and exclusion criteria.

**Figure 1:**
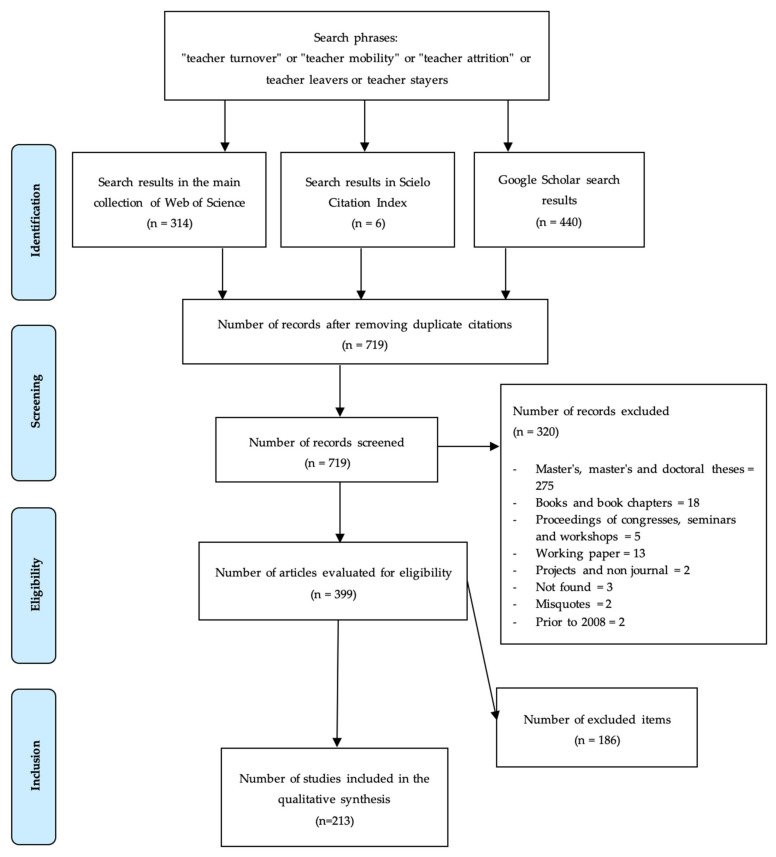
Flowchart of studies through the review.

In this process, 184 studies were excluded because they did not meet the inclusion criteria and 2 for other reasons (1 study was not identified in full and 1 study was excluded because it corresponded to grey literature, i.e., literature that does not need to be peer-reviewed to be published). Therefore, 213 studies were selected for qualitative synthesis of results. Figure 1 summarizes these processes.

### 2.6. Data Analysis Procedure

The selection of articles from each of the phases was systematized in an Excel table containing the following information: number (identifier code assigned to the document), source (source database), year of publication, journal, title, abstract, country, or geographical area of the research, keywords, and executive summary.

The results of each reviewer according to each of the phases were compared to identify coincidences and discrepancies. When disagreements were found among reviewers (CP and FN), the documents were reviewed again until an agreement was reached.

The complete reading of the articles selected in the last phase allowed the content analysis and the classification of themes to answer the research questions.

We built a data table including each read document with a unique identifying code and added the information derived from the reviewing process. Specifically, the extracted information synthesized and analyzed from the documents was: objective, methodology of the study, population and sample, stage of the teaching career studied, outcome, variables, instruments, types of analysis, results, and conclusions.

## 3. Results

### 3.1. Descriptive Results

**Figure 2:**
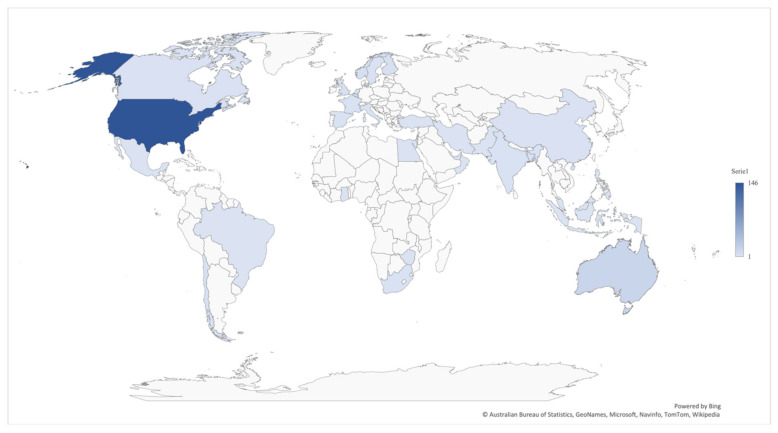
World map with frequency of publications on teacher mobility identified in the scoping review. Note: The image was created with the Excel tool powered by Bing and developed by Australian Bureau of Statistics, GeoNames, Microsoft, Navinfo, TomTom, and Wikipedia.

The descriptive results of the selection of studies (*n* = 213) show that most of the documents were research articles (95%), and only 5% corresponded to state documents or technical reports. Moreover, we identified 35 distinct countries of origin for the selected documents. Out of the total, 65% of the documents were from the United States (*n* = 146), followed in second place by Australia, with a percentage of 7% (*n* = 15). These results indicated there are more published studies of teacher mobility from North America, which was an expected trend, given that the high turnover rates in the United States are widely known [4,10]. Figure 2 illustrates the frequency of found studies per country. Countries with a more intense color produced more studies during these ten years (2008–2018), while countries with lighter colors showed a lower studies production. Countries in white represent countries with no published studies on teacher mobility.

**Figure 3:**
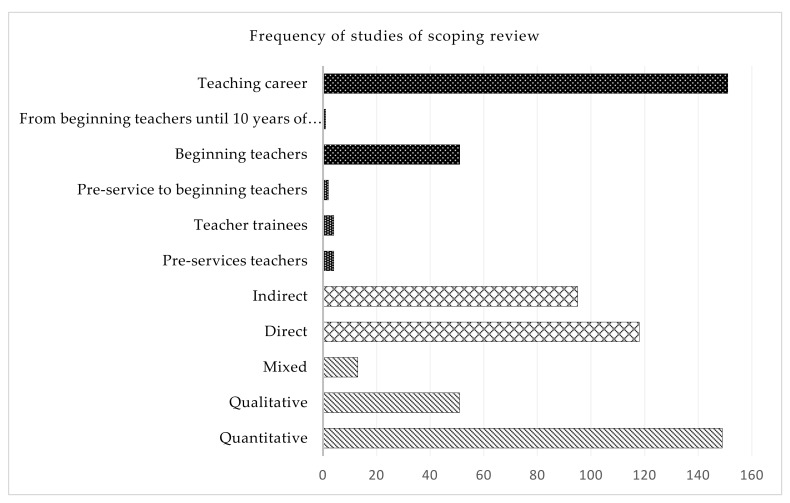
Frequency of identified studies of the scoping review according to race stage, perspective, and approach.

Regarding the approach used in the studies, it was identified that 70% (*n* = 149) used a quantitative approach, followed by a qualitative approach with 24% (*n* = 51). The least used approach is the mixed approach, with only 6% (*n* = 13) of the studies reviewed. We have identified two trends in this scoping review regarding the perspective used. A total of 55% (*n* = 118) of the literature approaches this phenomenon directly, while 45% (*n* = 95) approaches it indirectly. Regarding the addressed period of the teaching trajectory, where the studies were focused, we found six studies focused on the period prior to teaching, focused on pre-service teachers (3%). Four studies (2%) inquiry the teaching career period of practice, and 52 studies focused on beginning or novice teachers (24%), defined as those with five years or less experience. The remaining 151 studies (71%) did not focus on any period in specific, capturing all the periods their data could cover. Figure 3 displays the frequency of the identified studies according to the approach, perspective, and race stage of the scoping review. Details about the characteristics already mentioned and others such as source, year, journal, authors, and title are given in Table S2 of the Supplementary Materials.

### 3.2. Conceptualization and Operationalization of Teacher Mobility

About how teacher mobility is understood, that is, teacher turnover and attrition, which was the first question posed, it is essential to point out that we did not find studies that conceptually discuss a definition. The studies reviewed define turnover or attrition from multiple perspectives depending on how they operationalize it. Therefore, we have identified two operationalization trends that we have conceptually defined as “indirect mobility” and “direct mobility.”

Indirect mobility consists of the teacher rotation or abandonment that could occur in the future. Commonly, this approach involves studying the intention to work behavior, such as the intention to persist in the job post, the intention to leave the current job, and the intention to leave the profession. This approach often includes predictors more commonly found in organizational behavior studies, such as job satisfaction, organizational commitment, burnout, emotional exhaustion, among others. Indirect mobility, in this sense, is focused on the phenomenon of teacher mobility before the event of turnover or attrition occurs. In this classification, direct mobility includes studies that observed the events of teachers changing between schools and teachers leaving the profession (attrition). Using the present distinction between direct and indirect approaches to the study of teacher mobility, we found that 55% (*n* = 118) of the literature are direct studies. While 45% (*n* = 95) of the studies use indirect approaches, addressing teacher turnover before it has occurred. In summary, the study of teacher trajectories includes different teacher mobility forms, such as changes between schools and teachers (turnover) abandoning the teaching profession (attrition). Moreover, complementary, this research program includes the research of intentions of these different work behaviors, encompassing the period before the event of turnover and before the event of attrition. Therefore, teacher mobility is a phenomenon that can occur directly or indirectly and must be studied in all its complexity to propose actions that contribute to its reduction.

As already mentioned, the most widely used method to address teacher mobility is the quantitative approach, and least used is the mixed approach, a mixture of quantitative and qualitative methods. This figure was also an expected trend, given that there are numerous quantitative population-based studies [5,6,15], and this trend is reflected both in Chile and abroad [10,29,39,40,41].

Another important finding related to the first question of this review was detecting that direct mobility studies, apart from being the most frequent among the studies, commonly did not distinguish between voluntary (resignations) and involuntary lay-off (being fired). That is, it was not possible to distinguish those teachers who rotated or left of their own free will, resigning to change to another institution or to dedicate themselves to other work, from those teachers who were dismissed. This previous distinction is a significant omission in the study of teacher retention since the findings are often interpreted assuming that mobility is intentional or without delving into this very relevant aspect [42,43,44].

The previous omission is problematic because distinguishing between intentional and unintentional mobility makes it possible to understand two distinct types of teacher mobility, driven by different factors and sensitive to different interventions. For example, intentional mobility could be driven by more organizational aspects of the school and call for actions on attributes of the work environment such as leadership, workload, ad other stressing factors. In contrast, more structural factors could drive unintentional mobility among teachers. Dismissals or non-continuity of employment in establishments is a situation often caused by the modality of the system. In conclusion, distinguishing between intentional and non-intentional teacher mobility is necessary to design adequate actions for teacher retention efforts.

We can further distinguish the studies from the direct approach to teacher mobility regarding their time length. There are studies using a short period to address teacher mobility, for example, one year to the next, including Hanushek and Rivkin [45] or Ingersoll and May [46]. Furthermore, there are also studies employing longitudinal studies and panel studies following teacher mobility for many years. Examples of this latter class are Quartz [47], Elfers, Plecki and Van Windekens [48], and Donaldson and Johnson [49]. Most studies directly measuring mobility are almost cross-sectional [50,51,52,53,54], in the sense that they report those who stayed and those who left using a short period of observation. Moreover, we identified a group of studies that took both a direct and indirect approach, as they followed teachers from their tenure until they became mobile, such as the studies by Hong [55], DeAngelis, Wall, and Che [56], Lana [57] or Rinke and Mawhinney [58].

### 3.3. Factors Associated with Teacher Mobility

To answer the second question of this scoping review, about which factors are related to mobility, we organized the collected information from each study into five groups of covariates: (i) sociodemographic, such as biological and sociocultural factors of the population under study; (ii) those related to the teaching staff; (iii) those related to the school; (iv) working conditions, that is, those derived from the employment relationship; and, (v) organizational characteristics, that is, the specific conditions derived from the professional work in the organization where the teacher works [59]. The details of the factors identified can be found in Table 2 and are indicated below.

#### 3.3.1. Socio-Demographic Factors

Studies using quantitative methods provide more information on sociodemographic factors than the rest. Quantitative studies articles agree that mobility is more frequent in young teachers [40,42,77], male [170,183], belonging to urban environments [43], with characteristics of greater poverty or who serve more disadvantaged students [68], and who belong to isolated or remote localities [39]. Within the studies with a qualitative or mixed focus, there is an agreement that more disadvantage conditions seem to be related to greater mobility [3,197].

#### 3.3.2. Teacher-Related Factors

Studies with quantitative, qualitative, and mixed approaches agree that short experience is crucial in mobility [19,41,194,237]. This latter idea is consistent with many studies focusing on junior teachers or teachers with five years or less experience found in this review. Another teacher-related factor offered by the studies with a quantitative focus is whether teachers work in science mathematics disciplines [80,163,188], foreign languages [158], or special education [40]. Regarding certification in the teaching career, it seems that its absence is also a related factor [80,122]. However, studies along these lines qualify this information by relating it to lower or higher than average teacher certifications [240].

Furthermore, mobility is a phenomenon that occurs more frequently in teachers who work in secondary education [19,178]. Studies with a qualitative and mixed approach explain it as a lack of emotional gratification for teaching [3], intrinsic demotivation, or a lack of resilience [208]. Among the factors that stand out in studies with a qualitative approach, the determinants identified were the change in job orientation or the role that motivated the teaching career [199] or changes in the teachers’ perception of their identity [207,210].

#### 3.3.3. Student-Related Factors

The absence of studies with a qualitative approach that focuses on explaining mobility according to student factors is noteworthy. However, studies with a quantitative and mixed approach agree that the contexts with students from worse socio-economic or social conditions are key characteristics [46,125]. Additionally noteworthy are studies that have found higher teacher mobility when the students are from an ethnic minority, obtain lower grades in school, and exhibit more disruptive behavior [3,80,141,163,240].

#### 3.3.4. Working Conditions

One of the conditions for a more comprehensive agreement is the relationship between salaries and teacher mobility. Lower salaries are associated with higher rates of teacher mobility [69,85,96,98]. In this same group, factors such as the long working hours involved in the teaching profession [51,115,183,241] and having temporary contracts [73] stood out. Moreover, a lack of job placement or induction programs [30,194,235] and the type of schools with public administration, characterized by their systems and specific salary policies [124,134,137], were associated with greater teacher mobility.

#### 3.3.5. Organizational Conditions

One of the most evident conditions related to teacher turnover or abandonment, present in the reviewed articles, is excessive workload [134,144,195,196,209,242]. This factor is present in both quantitative and qualitative approach studies. A similar line of research consists of studies including emotional exhaustion, depersonalization, or detachment from the job, which are symptoms of burnout [53,79,133,156,191]. Complementary, some studies attribute this relationship, between workload and teacher mobility, as a consequence of stress [95,195,196]. All approaches, quantitative, qualitative, and mixed studies, agree that lack of support is positively related to teacher mobility. This lack of support can come from the leadership, the administration, or management [3,44,66,106,203], could be presented among co-workers [204,216] and results as determining factors in teacher mobility. Among organizational conditions, low job satisfaction [72,80,106,118,144,242] and low commitment [125,215,234,239] were also determinants of teacher mobility.

Regarding organizational factors, specifically personal resources, we found in the reviewed articles that teachers with lower self-efficacy were more likely to rotate or leave [40,154,162,196,224], along with those with less autonomy [46,225]. These latter sets of results are relevant given that specific actions of the work organization, such as encouraging participation in teacher training, could improve teacher self-efficacy and thus promote teacher retention. In the same way, actions such as granting teachers autonomy in their work could contribute to retention.

In summary, we have synthesized how it is teacher mobility is understood conceptually and how their research is approached. Moreover, we have summarized the common factors related to teacher mobility, the main trends presented across approaches, and its main agreements. The reviewed articles include a multiplicity of factors, thus affirming the multifactorial nature of this phenomenon. We distinguished two major perspectives in studying teacher mobility to aid further research: indirect and direct approaches. That is, studies addressing the intentions to leave schools or the profession and studies addressing teacher turnover and attrition. Making this latter distinction helps to understand and specify how the different factors covered by the literature are related to teacher mobility. With this scoping review, it is also possible to recognize the advantages and limitations of these different research approaches. For example, studies using prospective longitudinal design stood out. These studies can include indirect measures of teacher mobility (intentions to leave), thus addressing the event of leaving schools before it happens, while addressing teacher mobility directly (rotation or leaving as such), following teachers until the event has occurred. Although these studies were the least frequent, they were highly relevant since they can also distinguish between intentional and nonintentional mobility. While it is true that the approaches through which teacher mobility is studied are different, certain convergence was identified regarding the explanatory variables. Finally, we have proposed a comprehensive definition of teacher mobility to recognize the phenomenon’s complexity that could be useful.

## 4. Discussion and Conclusions

This review described the multiple ways of defining and measuring teacher mobility. For this, a definition of the phenomenon was proposed that recognizes two perspectives that summarize mobility’s conceptual and operational findings. These two perspectives approach teacher turnover and attrition indirectly and directly. The first perspective identifies the factors or conditions associated with teacher mobility at a particular future point in time. It is concerned with the willingness to leave the teaching job or profession and predictors expected to lead to such behavior. The second perspective is the direct approach and is responsible for identifying the factors or conditions associated with teacher mobility when the event has occurred. That is, its inquiries teachers who have already rotated or left. The direct perspective is the most frequent in the study of mobility, in which there are, in turn, two tendencies in its operationalization: on the one hand, those who measure turnover or abandonment in a short-term manner, and on the other, those who measure it longitudinally or in the long term. These two identified tendencies are consistent with the research designs identified by Murnane, Singer, and Willet [243] and those of Holme, Jabbar, Germain, and Dinning [244], given that they coincide in recognizing that there are two ways of studying teacher mobility according to the temporality that is analyzed. This latter consideration is highly relevant since how teacher mobility is operationalized conditions what factors are considered relevant. The proposals for improvement and the actors who can carry them out will be derived from them.

In addition, we identified that although there are fewer indirect studies of mobility, there has been an increase in their number in recent years. This research growth may be due to the advantages that the indirect perspective of mobility has for generating intervention proposals for teacher retention [63,245], making it a growing line of research. One of the advantages of the study of indirect mobility is its ability to distinguish two opposing but frequent types of mobility: intentional and un-intentional mobility. Unfortunately, it is common to find studies that do not identify this aspect, which is highly relevant for determining organizational or labor market responsibilities. In other words, the findings from studies that distinguish between these types of mobility can help us better understand the reasons why teachers leave the teaching profession voluntarily. In this sense, identifying the factors associated with mobility is highly relevant. For example, a case of teachers who leave because they retire or are fired, or their contract is not renewed could be different from teachers who leave voluntarily. Confounding these types of teacher mobilities can result in the underestimation or severity of the risk factor of each case.

Furthermore, by distinguishing the type of mobility, it is possible to understand the reasons that push teachers to abandon their establishments or professions more profoundly. This latter scenario includes risk factors such as high workload, poor working conditions such as a low hourly load that translates into lower wages, fewer resources to support teaching, among others. Interventions tailored at changing these factors could be considered as more structural and require long-term monitoring. Reducing workload may require hiring more teachers and changing teacher salaries in public schools may require law and policy changes. In contrast, interventions tailored at organizational factors, including improving leadership, organizational support, fostering teamwork, and promoting collaboration networks among teachers, may not suffer such a burden. In public educational systems, interventions at the organizational level of schools may not require structural changes at the legal level. Some of these expected changes could be implemented by the school leadership and its teachers. In summary, the distinction between what explains teacher mobility between strands of research is relevant because their possible lines of intervention are derived from their focus.

A surprising finding is some articles reporting higher teacher mobility among highly qualified teachers. In the review of Borman and Dowling [10], teachers with higher qualifications are expected to present less teacher attrition. This exception of the rule, covered by these qualitative studies, indicates that the dropping out of highly qualified teachers and better-evaluated teachers is due to a lack of a challenging teaching career that fails at talent retention, with little opportunities for professional development [41,82,196,246].

Another trend identified was the inclination to use a quantitative approach in indirect and direct mobility perspectives. Additionally, there was a clear majority of North American studies over other regions of the world [18,52,83]. We classified the different factors related to teacher mobility according to their object, including faculty, students, working conditions, and organizational conditions. Among the personal factors (faculty], the most frequent and significant is the short experience of teachers. Teacher mobility is higher during the first years of the teaching career. This finding is consistent with the findings of international [4,10,19] and national studies conducted in Chile [3,5,9,15,241,247,248]. As such, some studies focus on the first five years of the teaching career [47,51,125,199].

We also found that socio economic conditions, vulnerability, or poverty of the school and the educational environment increase the probability of teachers rotating or leaving, consistent with previous studies [16,46]. The present finding is particularly worrying for highly unequal educational systems. For example, in Chile, teachers with greater social capital are less likely to work in more vulnerable schools [22]. This result means that teachers with more favorable socio-demographic characteristics and with greater cultural capital are more likely to work in schools with the same characteristics. Complementary, teachers with fewer cultural and social resources are more likely to work in public schools and more socioeconomically disadvantaged schools, where there are worse working conditions and higher rates of teacher mobility [249]. Thus, teacher mobility from more socioeconomically disadvantaged schools could create a vicious cycle. Educational systems with high segregation, such as Chile, are examples of this scenario. Students’ allocation to schools is conditioned by the additional economic and social contribution of parents and guardians to their children’s education [247,249,250]. Moreover, teachers’ educational and social capital is related to the school environments in which they work [22]. These two features, in time, perpetuate higher teacher mobility in more socioeconomically disadvantaged schools.

The scoping review of the literature also allowed us to recognize the variables that possibly have the most significant relationship to teacher mobility. In this sense, we tested the hypothesis that organizational variables seem to be associated to a greater extent with both indirect and direct turnover and attrition. The variable that we identified as the main personal resource was teacher self-efficacy. Studies agree that teachers with higher perceived self-efficacy are less likely to become mentally exhausted or leave teaching [251,252,253,254,255,256]. This finding is highly relevant to fostering teacher retention and aligns with studies that claim that more competent teachers are less likely to leave the profession [252]. This finding suggests that promoting relevant teacher training, that is, contributing to improving the competencies that teachers perceive as most necessary, could foster the retention of qualified teachers.

Moreover, evidence indicates that one of the most problematic dimensions of self-efficacy is classroom management [251]. It is essential to consider that teachers need to constantly strive to achieve an adequate learning environment in the classroom. Furthermore, more disorder in the classroom tends to be higher in more vulnerable contexts, with high rates of violence, or where there are usually many students per classroom [50].

Among the conditions related to work resources, we identified the importance of school leadership, satisfaction with salaries, and the perception of support from co-workers. Leadership is one of the key factors in avoiding teacher mobility. The role played by the principal, or the management team of a school can be a conditioning factor when it comes to generating commitment to the job [44,257,258], being a protective factor for teacher retention [259,260,261]. Finally, the main work demands identified were workload and indiscipline in the classroom. Both cause teachers to exert sustained mental or physical effort on the job and predict burnout and attrition [34,53,62].

The present review used a comprehensive definition of teacher mobility, enabling a broad overview of the different factors related to teacher retention at their schools. The main findings of the present review are consistent with previous systematic reviews [10,29,33].

Among the limitations of this study only ten years were reviewed. It would be necessary to broaden the search range to identify more research and identify more solid trends. Moreover, it would be relevant to qualify the evidence’s quality with an established tool for these purposes. Further review exercises could focus on a limited population, such as beginning teachers. Limiting the population of focus can aid the use of meta-analysis techniques to synthesize the effect of organizational factors on teacher mobility in a precise manner, contributing to recent findings [10,28]. Moreover, more work is needed regarding specific disciplines, historical periods, and teaching periods within the teaching career, points asserted by the literature as relevant [4,16,188].

Finally, we believe that it is fundamental to extend questions beyond why teachers move, to include where they are going. In other words, what happens to their career paths, and what helps them to return. This latter extension would enrich the understanding of teacher mobility, providing more clues for teaching retention at their school and in the profession.

## Figures and Tables

**Table 1:** Search strategy for the scoping review of teacher turnover and attrition.

Database	Syntax
Web of Science Core Collection	TOPIC: (“teacher mobility”) OR TOPIC: (“teacher turnover”) OR TOPIC: (“teacher attrition”) OR TOPIC: (teacher leavers) OR TOPIC: (teacher stayers) Timespan: 2008–2018. Indexes: SCI-EXPANDED, SSCI, A&HCI, ESCI. Results: 314
SciELO Citation Index	TOPIC: (“teacher mobility”) OR TOPIC: (“teacher turnover”) OR TOPIC: (“teacher attrition”) OR TOPIC: (teacher leavers) OR TOPIC: (teacher stayers) Timespan: 2008–2018. Indexes: SCIELO Results: 6
Google Scholar	“teacher mobility” or “teacher turnover” or “teacher attrition” or teacher leavers or teacher stayers. Specific interval: 2008–2018 Results: 440

**Table 2:** Factors explaining teacher mobility according to approach.

	Quantitative	Qualitative	Mixed
Teachers	- Short experience (5 years or less) - Scientific-mathematical disciplines, foreign languages, and special education - Have unofficial teacher certification (no degree) - Much higher or much lower grades of teaching qualification - Lower teaching qualifications (although there are discrepancies) - Perform at the high school level - Low level of effectiveness (fewer grades).	- Changes in job orientation - Roles incongruent with the teaching job - Feeling of teaching incompetence - Intrinsic demotivation - Lack of adaptation - Loss of identity	- Short experience - Low sense of gratification for teaching work
Students	- Lower socioeconomic status - Greater difficulties with learning and lower performances in school - More disruptive behavior - Ethnic minorities		- Come from families with worse social and economic conditions - Have lower effectiveness rates (lower standardized test scores). - Ethnic minorities
Working conditions	- Low salary - High working hours - Short-term contracts - Type of school (administration)	- Low salary - High concentration of students per classroom	- Lack of induction - Type of school (administration)
Organizational	- Excessive workload - Low perception of self-efficacy - Emotional exhaustion, depersonalization, or detachment from the job (burnout symptoms). - Insufficient administrative support - Poor school leadership	- Excessive workload - Low perception of self-efficacy - Low commitment - High levels of stress - Lack of support among teachers - Little flexibility - Low autonomy	- Lack of commitment - Poor administration - Low perception of self-efficacy - Lack of ongoing peer support - Lack of leadership
References	[19,24,39,40,41,42,43,44,46,47,48,49,53,54,56,60,61,62,63,64,65,66,67,68,69,70,71,72,73,74,75,76,77,78,79,80,81,82,83,84,85,86,87,88,89,90,91,92,93,94,95,96,97,98,99,100,101,102,103,104,105,106,107,108,109,110,111,112,113,114,115,116,117,118,119,120,121,122,123,124,125,126,127,128,129,130,131,132,133,134,135,136,137,138,139,140,141,142,143,144,145,146,147,148,149,150,151,152,153,154,155,156,157,158,159,160,161,162,163,164,165,166,167,168,169,170,171,172,173,174,175,176,177,178,179,180,181,182,183,184,185,186,187,188,189,190]	[9,30,31,50,51,52,57,58,191,192,193,194,195,196,197,198,199,200,201,202,203,204,205,206,207,208,209,210,211,212,213,214,215,216,217,218,219,220,221,222,223,224,225,226,227,228,229,230]	[3,32,55,129,231,232,233,234,235,236,237,238,239]

## Supplementary Materials

The following supporting information can be downloaded at: https://www.mdpi.com/article/10.3390/ijerph19042313/s1, Table S1: Preferred Reporting Items for: Teacher Mobility, What Is It, How Is It Measured, And What Factors Determine It? A Scoping Review (pp. 2–4). Table S2: Descriptive characteristics of the publications on: Teacher Mobility, What Is It, How Is It Measured, And What Factors Determine It? A Scoping Review (pp. 5–25).
