# Supplementary material for: Teacher Mobility: What Is It, How Is It Measured and What Factors Determine It? A Scoping Review
Source: Int J Environ Res Public Health. 2022 Feb 17;19(4):2313. doi: 10.3390/ijerph19042313 (PMC8871816; doi:10.3390/ijerph19042313)
Supplement: Supplementary file 1 [file ijerph-19-02313-s001.zip › ijerph-1559504-supplementary.pdf]

Supplementary Files to “Teacher Mobility, What Is It, How Is It Measured, And What Factors Determine It? A Scoping Review”

By Claudia Palma-Vasquez [1,2,*], Diego Carrasco [3] and Mónica Tapia-Ladino [1]

[1] Faculty of Education, Universidad Católica de la Santísima Concepción, Concepción 4090541, Chile.
[2] Center for Research in Occupational Health (CiSAL), Department of Experimental and Health Sciences, Pompeu Fabra University, Barcelona 08003, Spain.
[3] Pontificia Universidad Católica de Chile, Centro de Medición MIDE UC. Santiago 7820436, Chile.
* Correspondence: cpalma@doctoradoedu.ucsc.cl

Index:

- Supplementary Material, Table S1: "Preferred Reporting Items for: Teacher Mobility, What Is It, How Is It Measured, And What Factors Determine It? A Scoping Review” (p. 2-4)
- Supplementary Material, Table S2: “Descriptive characteristics of the publications on: Teacher Mobility, What Is It, How Is It Measured, And What Factors Determine It? A Scoping Review” (p. 5-25)

Table S1: Preferred Reporting Items for: Teacher Mobility, What Is It, How Is It Measured, And What Factors Determine It? A Scoping Review

Note: From the Systematic reviews and Meta-Analyses extension for Scoping Reviews (PRISMA-ScR) Checklist (Tricco et al., 2018)

SECTION	ITEM	PRISMA-ScR CHECKLIST ITEM	REPORTED ON PAGE #
TITLE			
Title	1	Identify the report as a scoping review.	page 1 (line 2)
ABSTRACT			
Structured summary	2	Provide a structured summary that includes (as applicable): background, objectives, eligibility criteria, sources of evidence, charting methods, results, and conclusions that relate to the review questions and objectives.	page 1 (lines 10-33)
INTRODUCTION			
Rationale	3	Describe the rationale for the review in the context of what is already known. Explain why the review questions/objectives lend themselves to a scoping review approach.	page 2 (lines 91-104)
Objectives	4	Provide an explicit statement of the questions and objectives being addressed with reference to their key elements (e.g., population or participants, concepts, and context) or other relevant key elements used to conceptualize the review questions and/or objectives.	page 2 (lines 104-107)
METHODS			
Protocol and registration	5	Indicate whether a review protocol exists; state if and where it can be accessed (e.g., a Web address); and if available, provide registration information, including the registration number.	An internal protocol was developed to document the criteria for do this scoping review but this is not published.
Eligibility criteria	6	Specify characteristics of the sources of evidence used as eligibility criteria (e.g., years considered, language, and publication status), and provide a rationale.	page 3 (lines 124-137)
Information sources*	7	Describe all information sources in the search (e.g., databases with dates of coverage and contact with authors to identify additional sources), as well as the date the most recent search was executed.	page 3 (lines 138-142)
Search	8	Present the full electronic search strategy for at least 1 database, including any limits used, such that it could be repeated.	page 3-4 (lines 143-146)
Selection of sources of evidence†	9	State the process for selecting sources of evidence (i.e., screening and eligibility) included in the scoping review.	pages 3-4 (lines 108-174)
Data charting process‡	10	Describe the methods of charting data from the included sources of evidence (e.g., calibrated forms or forms that have been tested by the team before their use, and whether data charting was done independently or in	page 5 (lines 175-176)

SECTION	ITEM	PRISMA-ScR CHECKLIST ITEM	REPORTED ON PAGE #
		duplicate) and any processes for obtaining and confirming data from investigators.	
Data items	11	List and define all variables for which data were sought and any assumptions and simplifications made.	page 6 (lines 188-191)
Critical appraisal of individual sources of evidence§	12	If done, provide a rationale for conducting a critical appraisal of included sources of evidence; describe the methods used and how this information was used in any data synthesis (if appropriate).	page 5-6 (lines 178-191)
Synthesis of results	13	Describe the methods of handling and summarizing the data that were charted.	Table S2 (Supplementary Materials)
RESULTS			
Selection of sources of evidence	14	Give numbers of sources of evidence screened, assessed for eligibility, and included in the review, with reasons for exclusions at each stage, ideally using a flow diagram.	page 6 (lines 193-203)
Characteristics of sources of evidence	15	For each source of evidence, present characteristics for which data were charted and provide the citations.	page 10 (lines 296)
Critical appraisal within sources of evidence	16	If done, present data on critical appraisal of included sources of evidence (see item 12).	no data
Results of individual sources of evidence	17	For each included source of evidence, present the relevant data that were charted that relate to the review questions and objectives.	page 7 (lines 209-224, figure 3) page 7 (lines 226-294, table 2) page 11 (lines 297-371)
Synthesis of results	18	Summarize and/or present the charting results as they relate to the review questions and objectives.	page 6 (lines 205-207, figure 2) page 7 (lines 223-225, figure 3) page 10 (lines 295-296, table 2)
DISCUSSION			
Summary of evidence	19	Summarize the main results (including an overview of concepts, themes, and types of evidence available), link to the review questions and objectives, and consider the relevance to key groups.	page 12 (lines 373-480)
Limitations	20	Discuss the limitations of the scoping review process.	page 12-14 (lines 481-487)
Conclusions	21	Provide a general interpretation of the results with respect to the review questions and objectives, as well as potential implications and/or next steps.	page 12-14 (lines 481-487)
FUNDING			
Funding	22	Describe sources of funding for the included sources of evidence, as well as sources of	page 14 (lines 507-508)

SECTION	ITEM	PRISMA-ScR CHECKLIST ITEM	REPORTED ON PAGE #
		funding for the scoping review. Describe the role of the funders of the scoping review.	

JBI = Joanna Briggs Institute; PRISMA-ScR = Preferred Reporting Items for Systematic reviews and Meta-Analyses extension for Scoping Reviews.
* Where sources of evidence (see second footnote) are compiled from, such as bibliographic databases, social media platforms, and Web sites.
† A more inclusive/heterogeneous term used to account for the different types of evidence or data sources (e.g., quantitative and/or qualitative research, expert opinion, and policy documents) that may be eligible in a scoping review as opposed to only studies. This is not to be confused with information sources (see first footnote).
‡ The frameworks by Arksey and O'Malley (6) and Levac and colleagues (7) and the JBI guidance (4, 5) refer to the process of data extraction in a scoping review as data charting.
§ The process of systematically examining research evidence to assess its validity, results, and relevance before using it to inform a decision. This term is used for items 12 and 19 instead of "risk of bias" (which is more applicable to systematic reviews of interventions) to include and acknowledge the various sources of evidence that may be used in a scoping review (e.g., quantitative and/or qualitative research, expert opinion, and policy document).

Reference:

Tricco, A. C., Lillie, E., Zarin, W., O'Brien, K. K., Colquhoun, H., Levac, D., . . . Weeks, L. (2018). PRISMA extension for scoping reviews (PRISMA-ScR): checklist and explanation. *Annals of internal medicine, 169*(7), 467-473. https://doi.org/https://doi.org/10.7326/M18-0850

Table S2. Descriptive characteristics of the publications on: Teacher Mobility, What Is It, How Is It Measured, And What Factors Determine It? A Scoping Review

ID	Source	Year	Type	Magazine	Authors	Title	Country	Approach	Perspective	Race stage
1	WoS CC	2016	Article	Modern Language Journal	Acheson K. Taylor J. & Luna K.	The Burnout Spiral: The Emotion Labor of Five Rural US Foreign Language Teachers	USA	Qualitative	Indirect	Teaching career
2	WoS CC	2017	Article	Educational Evaluation and Policy Analysis	Adnot M. Dee T. Katz V. & Wyckoff J.	Teacher Turnover Teacher Quality and Student Achievement in DCPS	USA	Quantitative	Direct	Teaching career
3	WoS CC	2015	Article	Cambridge Journal of Education	Adoniou M.	'It's very much taken as an insult if I say anything': do new educators have a right to speak their mind?	Australia	Qualitative	Indirect	Beginning Teachers
4	GS	2010	Article	International journal of education reform	Agezo K.	Why teachers leave teaching: The case of pre-tertiary institutions in Ghana.	Ghana	Qualitative	Direct	Beginning Teachers
5	WoS CC	2016	Article	Questions Vives-Questions in Education	Alava S.	Teacher facing the difficulty of class: disempowerment and teacher attrition	France	Quantitative	Indirect	Teaching career
6	WoS CC	2016	Article	Ra Ximhai Magazine	Alegria-Rivas L. M. E.	School violence: the practice teachers and school leaving.	Mexico	Qualitative	Indirect	Teaching career
7	WoS CC	2018	Article	Education Economics	Allen R. Burgess S. & Mayo J.	The teacher labour market teacher turnover and disadvantaged schools: new evidence for England.	England	Quantitative	Direct	Teaching career
8	WoS CC	2014	Article	Urban Education	Aragon A. Culpepper S. A. McKee M. W. & Perkins M.	Understanding Profiles of Preservice Teachers With Different Levels of Commitment to Teaching in Urban Schools.	USA	Quantitative	Indirect	Teacher trainees
9	WoS CC	2016	Article	Australian Journal of Education	Arnup J. & Bowles T.	Should I stay or should I go? Resilience as a protective factor for teachers' intention to leave the teaching profession.	Australia	Quantitative	Indirect	From Beginning Teachers until 10 years of experience
10	WoS CC	2016	Article	International Journal of Educational Development	Avalos B. & Valenzuela J. P.	Education for all and attrition/retention of new teachers: A trajectory study in Chile.	Chile	Mixed (quantitative and qualitative)	Direct	Beginning Teachers
11	GS	2009	Article	American Politics	Balu R. Béteille T. & Loeb S.	Examining teacher turnover: The role of school leadership.	USA	Quantitative	Direct	Teaching career

12	WoS CC	2011	Article	Economics of Education Review	Barbieri G. Rossetti C. & Sestito P.	The determinants of teacher mobility: Evidence using Italian teachers' transfer applications.	Italy	Quantitative	Indirect	Teaching career
13	GS	2014	Report/ Technical Report	National Institute for Excellence in Teaching	Barnett J. H. & Hudgens T. M.	Staying Power: The Impact of the TAP System on Retaining Teachers Nationwide.	USA	Quantitative	Direct	Teaching career
14	GS	2014	Article	Education	Battle A. & Looney L.	Teachers' intentions to stay in teaching: The role of values and knowledge of adolescent development.	USA	Mixed (quantitative and qualitative)	Indirect	Teaching career
15	WoS CC	2014	Article	Qualitative Inquiry	Beaton J.	Perceiving Professional Risk in Five Stories.	USA	Qualitative	Indirect	Beginning Teachers
16	WoS CC	2017	Article	Remedial and Special Education	Bettini E. Jones N. Brownell M. Conroy M. Park Y. Leite W. Crockett J. Benedict A.	Workload Manageability Among Novice Special and General Educators: Relationships With Emotional Exhaustion and Career Intentions.	USA	Quantitative	Indirect	Beginning Teachers
17	GS	2012	Article	Georgia Educational Researcher	Bhatnagar R. Kim J. & Many J. E.	One Urban College of Education's Examination of Graduates' Employment and Retention in Public Schools.	USA	Quantitative	Direct	Teaching career
18	WoS CC	2008	Article	Exceptional Children	Boe E. E. E. Cook L. H. & Sunderland R. J.	Teacher turnover: Examining exit attrition teaching area transfer and school migration.	USA	Quantitative	Direct	Teaching career
19	WoS CC	2016	Article	Economic Journal	Bonhomme S. Jolivet G. & Leuven E.	School Characteristics and Teacher Turnover: Assessing the Role of Preferences and Opportunities.	Netherlands	Quantitative	Direct	Teaching career
20	GS	2009	Report/ Technical Report	Mathematica Policy Research Inc.	Booker K. & Glazerman S.	Effects of the Missouri Career Ladder Program on Teacher Mobility.	USA	Quantitative	Direct	Teaching career
21	GS	2013	Article	Leadership and Policy Quarterly	Bower C. B.	Teacher Retention and School Performance in High-Poverty Urban Schools: Evidence from New York City Middle Schools.	USA	Quantitative	Direct	Teaching career
22	GS	2011	Article	American Educational Research Journal	Boyd D. Grossman P. Ing M. Lankford H. Loeb S. & Wyckoff J.	The influence of school administrators on teacher retention decisions.	USA	Quantitative	Direct	Teaching career
23	GS	2013	Article	Electronic Journal of Science Education	Bozeman T. D. Scoggin S. & Stuessy C.	Job satisfaction of high school science teachers: Prevalence and association with teacher retention.	USA	Quantitative	Direct	Teaching career
24	WoS CC	2012	Article	Economic Record	Bradley S. Green C. & Mangan J.	The Effect of Relative Wages and External Shocks on Public Sector Turnover.	Australia	Quantitative	Direct	Teaching career

25	WoS CC	2017	Article	Educational Policy	Brummet Q. Gershenson S. & Hayes M. S. (2017).	Teachers' Grade-Level Reassignments: Evidence From Michigan.	USA	Quantitative	Direct	Teaching career
26	WoS CC	2016	Article	Questions Vives-Recherches En Education	Bruno F. (2016).	Prendre en compte les élèves à risque de décrochage scolaire au sein du cours disciplinaire : une prise de risque pour les enseignants	France	Quantitative	Indirect	Teaching career
27	WoS CC	2010	Article	Asia Pacific Journal of Education	Buchanan J. (2010).	May I be excused? Why teachers leave the profession.	Australia	Qualitative	Direct	Teaching career
28	WoS CC	2012	Article	Australian Journal of Education	Buchanan J. (2012).	Telling tales out of school: Exploring why former teachers are not returning to the classroom.	Australia	Qualitative	Direct	Teaching career
29	GS	2015	Article	Teaching and Teacher Education	Burke P. F. Aubusson P. J. Schuck S. R. Buchanan J. D. & Prescott A. E. (2015)	How do early career teachers value different types of support? A scale-adjusted latent class choice model.	Australia	Quantitative	Indirect	Beginning Teachers
30	WoS CC	2017	Article	Educational Evaluation and Policy Analysis	Burkhauser S. (2017).	How Much Do School Principals Matter When It Comes to Teacher Working Conditions?	USA	Quantitative	Indirect	Teaching career
31	WoS CC	2016	Article	In Education	Burleigh D. (2016).	Teacher Attrition in a Northern Ontario Remote First Nation: A Narrative Re-Storying.	Canada	Qualitative	Indirect	Teaching career
32	GS	2013	Article	SSRN	Calimeris L. (2013).	Teacher Attrition in Charter and Public Schools.	USA	Quantitative	Direct	Teaching career
33	WoS CC	2013	Article	Education and Treatment of Children	Cancio E. J. Albrecht S. F. & Johns B. H. (2013).	Defining Administrative Support and Its Relationship to the Attrition of Teachers of Students with Emotional and Behavioral Disorders.	USA	Quantitative	Indirect	Teaching career
34	WoS CC	2010	Article	Journal of Health Psychology	Carson R. L. Baumgartner J. J. Matthews R. A. & Tsouloupas C. N. (2010)	Emotional Exhaustion Absenteeism and Turnover Intentions in Childcare Teachers Examining the Impact of Physical Activity Behaviors	USA	Quantitative	Indirect	Teaching career
35	GS	2017	Report/ Technical Report	Learning Policy Institute	Carver-Thomas D. & Darling-Hammond L. (2017).	Teacher turnover: Why it matters and what we can do about it.	USA	Quantitative	Direct	Teaching career

36	Scielo CI	2014	Article	Education & Society	Cassettari N. Scaldelai V. D. F. & Frutuoso P. C. (2014).	Exoneração a pedido de professores: estudo em duas redes municipais paulistas	Brazil	Quantitative	Direct	Teaching career
37	WoS CC	2010	Article	Teaching and Teacher Education	Castro A. J. Kelly J. & Shih M. (2010).	Resilience strategies for new teachers in high-needs areas.	USA	Qualitative	Indirect	Beginning Teachers
38	WoS CC	2011	Article	School Effectiveness and School Improvement	Cha S. H. & Cohen-Vogel L. (2011).	Why they quit: a focused look at teachers who leave for other occupations.	USA	Quantitative	Direct	Teaching career
39	WoS CC	2018	Article	International Journal of Public Administration	Choi Y. & Chung I. H. (2018).	Effects of Public Service Motivation on Turnover and Job Satisfaction in the US Teacher Labor Market.	USA	Quantitative	Indirect	Teaching career
40	WoS CC	2016	Article	European Journal of Teacher Education	Christophersen K. A. Elstad E. Solhaug T. & Turmo A. (2016).	Antecedents of student teachers' affective commitment to the teaching profession and turnover intention.	Norway	Quantitative	Indirect	Teacher trainees
41	WoS CC	2015	Article	Teaching and Teacher Education	Clandinin D. Long J. Schaefer L. Downey C. A. Steeves P. Pinnegar E. Robblee S. M. & Wnuk S.	Early career teacher attrition: intentions of teachers beginning.	Canada	Qualitative	Indirect	Beginning Teachers
42	WoS CC	2017	Article	Teaching and Teacher Education	Clara M. (2017).	Teacher resilience and meaning transformation: How teachers reappraise situations of adversity.	Spain	Qualitative	Indirect	Teaching career
43	WoS CC	2011	Article	Education Finance and Policy	Clotfelter C. T. Ladd H. F. & Vigdor J. L. (2011).	Teacher mobility school segregation and pay-based policies to level the playing field.	USA	Quantitative	Direct	Teaching career
44	WoS CC	2012	Article	American Educational Research Journal	Cochran-Smith M. McQuillan P. Mitchell K. Terrell D. G. Barnatt J. D'Souza L. Jong C. Shakman K. Lam K. & Gleeson A. M.	A Longitudinal Study of Teaching Practice and Early Career Decisions: A Cautionary Tale.	USA	Qualitative	Direct	Beginning Teachers
45	WoS CC	2012	Article	Economics of Education Review	Cowen J. M. Butler J. S. Fowles J. Streams M. E. & Toma E. F. (2012).	Teacher retention in Appalachian schools: Evidence from Kentucky.	USA	Quantitative	Direct	Teaching career

46	WoS CC	2013	Article	Education Finance and Policy	Cowen J. M. & Winters M. A. (2013).	Do charters retain teachers differently? evidence from elementary schools in Florida.	USA	Quantitative	Direct	Teaching career
47	WoS CC	2014	Article	Journal of Curriculum Studies	Craig C. J. (2014).	From stories of staying to stories of leaving: a US beginning teacher's experience.	USA	Qualitative	Direct	Beginning Teachers
48	GS	2012	Article	International Scholarly Research Network (ISRN Education)	Curtis C. (2012).	Keeping Our Nation's Mathematics Teachers.	USA	Quantitative	Indirect	Teaching career
49	GS	2017	Article	Alberta Journal of Educational Research	Dahlkamp S. Peters M. L. & Schumacher G. (2017)	Principal Self-Efficacy School Climate and Teacher Retention: A Multi-Level Analysis.	USA	Quantitative	Indirect	Teaching career
50	WoS CC	2011	Article	Education and Urban Society	DeAngelis K. J. & Presley J. B. (2011).	Toward a More Nuanced Understanding of New Teacher Attrition.	USA	Quantitative	Direct	Teaching career
51	GS	2013	Article	Education Policy Analysis Archives	DeAngelis K. J. (2013)	A look at returning teachers.	USA	Quantitative	Direct	Teaching career
52	WoS CC	2013	Article	Journal of Teacher Education	DeAngelis K. J. Wall A. F. & Che J. (2013).	The Impact of Preservice Preparation and Early Career Support on Novice Teachers' Career Intentions and Decisions.	USA	Quantitative	Indirect	Pre-service to Beginning Teachers
53	GS	2017	Report/ Technical Report	Center for Alaska Education Policy Research	DeFeo D. J. Tran T. Hirshberg D. Cope D. & Cravez P. (2017).	The cost of teacher turnover in Alaska.	USA	Quantitative	Direct	Pre-services teachers
54	WoS CC	2016	Article	Learning and Teaching in Higher Education-Gulf Perspectives	Dickson M. McMinn M. & Kadbey H. (2016).	Science anxiety levels in Emirati student teachers.	United Arab Emirates	Quantitative	Indirect	Pre-services teachers
55	WoS CC	2009	Article	Perspectives in Education	Diko N. N. & Letseka M. (2009).	Policy appropriation in teacher retention and attrition: the case of North-West Province.	South Africa	Qualitative	Direct	Pre-service to Beginning Teachers
56	WoS CC	2016	Article	Race Ethnicity and Education	Djonko-Moore C. M. (2016).	An exploration of teacher attrition and mobility in high poverty racially segregated schools.	USA	Quantitative	Direct	Pre-services teachers
57	GS	2010	Article	Educational Evaluation and Policy Analysis	Donaldson M. L. & Johnson S. M. (2010).	The price of misassignment: The role of teaching assignments in Teach for America teachers' exit from low-income schools and the teaching profession.	USA	Quantitative	Direct	Beginning Teachers
58	WoS CC	2016	Article	International Journal of Educational Research	Donitsa-Schmidt S. & Zuzovsky R. (2016).	Quantitative and qualitative teacher shortage and the turnover phenomenon.	Israel	Quantitative	Direct	Teaching career

59	WoS CC	2018	Article	Education and Urban Society	Dunn A. H. & Downey C. A.	Betting the House: Teacher Investment Identity and Attrition in Urban Schools.	USA	Qualitative	Indirect	Teaching career
60	WoS CC	2016	Article	British Educational Research Journal	Dupriez V. Delvaux B. & Lothaire S. (2016).	Teacher shortage and attrition: Why do they leave?	Belgium	Quantitative	Direct	Beginning Teachers
61	GS	2017	Report/ Technical Report	University of Washington College of Education Center for the Study of Teaching and Policy	Elfers A. M. Plecki M. L. & Van Windekens A. (2017).	Understanding Teacher Retention and Mobility in Washington State.	USA	Quantitative	Direct	Teaching career
62	WoS CC	2011	Article	American Economic Review	Falch T. (2011).	Teacher Mobility Responses to Wage Changes: Evidence from a Quasi-Natural Experiment.	Norway	Quantitative	Direct	Teaching career
63	WoS CC	2018	Article	Urban Education	Farinde-Wu A. & Fitchett P. G. (2018).	Searching for Satisfaction: Black Female Teachers' Workplace Climate and Job Satisfaction.	USA	Quantitative	Indirect	Teaching career
64	WoS CC	2009	Article	Southern Economic Journal	Feng L. (2009).	Opportunity Wages Classroom Characteristics and Teacher Mobility.	USA	Quantitative	Direct	Teaching career
65	WoS CC	2010	Article	Education Finance and Policy	Feng L. (2010).	Hire today gone tomorrow: new teacher classroom assignments and teacher mobility.	USA	Quantitative	Direct	Beginning Teachers
66	WoS CC	2018	Article	Journal of Urban Economics	Feng L, Figlio D. & Sass T. (2018).	School accountability and teacher mobility.	USA	Quantitative	Direct	Teaching career
67	WoS CC	2012	Article	Andrew Young School of Policy Studies Research Paper Series	Feng L. & Sass T. (2012)	Teacher quality and teacher mobility.	USA	Quantitative	Direct	Teaching career
68	WoS CC	2017	Article	Education Finance and Policy	Feng L. & Sass T. R. (2017).	Teacher Quality and Teacher Mobility.	USA	Quantitative	Direct	Teaching career
69	GS	2011	Article	Alberta Journal of Educational Research	Fontaine S. Kane R. Duquette O. & Savoie-Zajc L. (2011).	New teachers' career intentions: Factors influencing new teachers' decisions to stay or to leave the profession	Australia	Mixed (quantitative and qualitative)	Indirect	Beginning Teachers

70	WoS CC	2009	Article	Journal of Teacher Education	Freedman S. W. & Appleman D. (2009)	"In It for the Long Haul" How Teacher Education Can Contribute to Teacher Retention in High-Poverty Urban Schools.	USA	Mixed (quantitative and qualitative)	Direct	Beginning Teachers
71	WoS CC	2017	Article	Reflective Practice	Frelin A. & Fransson G. (2017).	Four components that sustain teachers' commitment to students - a relational and temporal model.	Sweden	Qualitative	Indirect	Teaching career
72	WoS CC	2014	Article	Educational Evaluation and Policy Analysis	Fulbeck E. S. (2014).	Teacher Mobility and Financial Incentives: A Descriptive Analysis of Denver's ProComp.	USA	Quantitative	Direct	Teaching career
73	WoS CC	2015	Article	Teachers College Record	Fulbeck E. S. & Richards M. P. (2015).	The Impact of School-Based Financial Incentives on Teachers' Strategic Moves: A Descriptive Analysis.	USA	Quantitative	Direct	Teaching career
74	WoS CC	2016	Article	American Journal of Education	Fuller B. Waite A. & Irribarra D. T. (2016).	Explaining Teacher Turnover: School Cohesion and Intrinsic Motivation in Los Angeles.	USA	Quantitative	Indirect	Teaching career
75	Scielo CI	2017	Article	Pedagogical studies (Valdivia)	Gaete Silva A. Castro Navarrete M. Pino Conejeros F. & Mansilla Devia D. (2017).	Leaving the teaching profession in Chile: Reasons for leaving the classroom and conditions for returning.	Chile	Qualitative	Direct	Teaching career
76	WoS CC	2017	Article	Teachers and Teaching	Gallant A. & Riley P. (2017).	Early career teacher attrition in Australia: inconvenient truths about new public management.	Australia	Qualitative	Indirect	Beginning Teachers
77	WoS CC	2011	Article	Economics of Education Review	Gilpin G. A. (2011).	Reevaluating the effect of non-teaching wages on teacher attrition.	USA	Quantitative	Direct	Teaching career
78	WoS CC	2018	Article	Teachers and Teaching	Glazer J (2018).	Leaving lessons: learning from the exit decisions of experienced teachers.	USA	Qualitative	Direct	Teaching career
79	GS	2016	Article	Journal of Education and Training Studies	Glennie E. J. Mason M. & Edmunds J. A. (2016).	Retention and satisfaction of novice teachers: Lessons from a school reform model.	USA	Quantitative	Direct	Beginning Teachers
80	WoS CC	2011	Article	Journal of Policy Analysis and Management	Goldhaber D. Gross B. & Player D. (2011)	Teacher Career Paths, Teacher Quality, and Persistence in the Classroom: Are Public Schools	USA	Quantitative	Direct	Beginning Teachers

81	WoS CC	2014	Article	Journal of Teacher Education	Goldhaber D. & Cowan J. (2014).	Excavating the Teacher Pipeline: Teacher Preparation Programs and Teacher Attrition.	USA	Quantitative	Direct	Teaching career
82	WoS CC	2015	Article	Educational Researcher	Goldhaber D. Grout C. Holden K. L. & Brown N. (2015).	Crossing the Border? Exploring the Cross-State Mobility of the Teacher Workforce.	USA	Quantitative	Direct	Teaching career
83	WoS CC	2016	Article	journal of Policy Analysis and Management	Goldhaber D. Lavery L. & Theobald R. (2016).	Inconvenient Truth? Do Collective Bargaining Agreements Help Explain the Mobility of Teachers within School Districts?	USA	Quantitative	Direct	Teaching career
84	WoS CC	2016	Article	Educational Evaluation and Policy Analysis	Goldhaber D. Strunk K. O. Brown N. & Knight D. S. (2016).	Lessons Learned From the Great Recession: Layoffs and the RIF-Induced Teacher Shuffle.	USA	Quantitative	Direct	Teaching career
85	WoS CC	2015	Article	International Journal of Instruction	Gomba C. (2015).	Why Do They Stay: Factors Influencing Teacher Retention in Rural Zimbabwe.	Zimbabwe	Qualitative	Direct	Teaching career
86	WoS CC	2016	Article	Educational Studies	Gottfried M. A. & Straubhaar R. (2015).	The perceived role of the Teach For America program on teachers' long-term career aspirations.	USA	Qualitative	Indirect	Beginning Teachers
87	WoS CC	2011	Article	Journal of Policy Analysis and Management	Grissom J. A. & Keiser L. R. (2011).	A Supervisor Like Me: Race Representation and the Satisfaction and Turnover Decisions of Public Sector Employees.	USA	Quantitative	Indirect	Teaching career
88	WoS CC	2011	Article	Teachers College Record	Grissom J. A. (2011).	Can Good Principals Keep Teachers in Disadvantaged Schools? Linking Principal Effectiveness to Teacher Satisfaction and Turnover in Hard-to-Staff Environments.	USA	Quantitative	Indirect	Teaching career
89	GS	2014	Article	Teachers and Teaching	Gu Q. (2014).	The role of relational resilience in teachers' career-long commitment and effectiveness.	England	Qualitative	Indirect	Teaching career
90	GS	2016	Article	Journal of Organizational and Educational Leadership	Gulosino C. Franceschini III L. & Hardman P. (2016).	The Influence of Balance Within the Competing Values Framework and School Academic Success on Teacher Retention.	USA	Quantitative	Indirect	Teaching career

91	WoS CC	2008	Article	Journal of Experimental Education	Hahs-Vaughn D. L. & Scherff L. (2008).	Beginning English teacher attrition mobility and retention. Journal of Experimental Education	USA	Quantitative	Indirect	Beginning Teachers
92	GS	2008	Article	Journal of Research in Music Education	Hancock C. B. (2008).	Music Teachers at Risk for Attrition and Migration: An Analysis of the 1999-2000 Schools and Staffing Survey.	USA	Quantitative	Indirect	Teaching career
93	WoS CC	2009	Article	Journal of Research in Music Education	Hancock C. B. (2009)	National Estimates of Retention Migration and Attrition A Multiyear Comparison of Music and Non-music Teachers.	USA	Quantitative	Direct	Teaching career
94	GS	2016	Article	Journal of Research in Music Education	Hancock C. B. (2016).	Is the grass greener? Current and former music teachers' perceptions a year after moving to a different school or leaving the classroom.	USA	Quantitative	Direct	Teaching career
95	WoS CC	2010	Article	Journal of Teacher Education	Hancock C. B. & Scherff L. (2010).	Who Will Stay and Who Will Leave? Predicting Secondary English Teacher Attrition Risk.	USA	Quantitative	Indirect	Teaching career
96	WoS CC	2016	Article	Educational Evaluation and Policy Analysis	Hansen M. Backes B. & Brady V. (2016).	Teacher Attrition and Mobility During the Teach for America Clustering Strategy in Miami-Dade County Public Schools.	USA	Quantitative	Direct	Teaching career
97	WoS CC	2016	Article	Economics of Education Review	Hanushek E. A. Rivkin S. G. & Schiman J. C. (2016).	Dynamic effects of teacher turnover on the quality of instruction.	USA	Quantitative	Direct	Teaching career
98	WoS CC	2015	Article	Journal of Teacher Education	Harfitt G. J. (2015).	From attrition to retention: a narrative inquiry of why beginning teachers leave and then rejoin the profession.	Hong Kong	Qualitative	Direct	Beginning Teachers
99	WoS CC	2014	Article	Urban Education	Heineke A. J. Mazza B. S. & Tichnor-Wagner A. (2014).	After the Two-Year Commitment: A Quantitative and Qualitative Inquiry of Teach For America Teacher Retention and Attrition	USA	Mixed (quantitative and qualitative)	Direct	Beginning Teachers
100	WoS CC	2014	Article	Journal of Public Economics	Hendricks M. D. (2014).	Does it pay to pay teachers more? Evidence from Texas. Journal of Public Economics	USA	Quantitative	Direct	Teaching career

101	GS	2015	Article	SSRN	Hendricks M. D. (2015).	Public Schools Are Hemorrhaging Talented Teachers: Can Higher Salaries Function as a Tourniquet?	USA	Quantitative	Direct	Teaching career
102	WoS CC	2011	Article	Educational Researcher	Henry G. T. Bastian K. C. & Fortner C. K. (2011).	Stayers and Leavers: Early-Career Teacher Effectiveness and Attrition.	USA	Quantitative	Direct	Beginning Teachers
103	WoS CC	2010	Article	Teaching and Teacher Education	Hong J. Y. (2010).	Pre-service and beginning teachers' professional identity and its relation to dropping out of the profession. Teaching and Teacher Education	USA	Mixed (quantitative and qualitative)	Direct	Teacher trainees
104	WoS CC	2012	Article	Teachers and Teaching	Hong J. Y. (2012)	Why do some beginning teachers leave the school and others stay? Understanding teacher resilience through psychological lenses.	USA	Qualitative	Direct	Beginning Teachers
105	WoS CC	2018	Article	Teaching and Teacher Education	Horvath M. Goodell J. E. & Kosteas V. D. (2018).	Decisions to enter and continue in the teaching profession: Evidence from a sample of US secondary STEM teacher candidates.	USA	Quantitative	Indirect	Beginning Teachers
106	WoS CC	2015	Article	Issues in Educational Research	Howes L. M. & Goodman-Delahunty J. (2015).	Teachers' career decisions: Perspectives on choosing teaching careers and on staying or leaving.	USA	Qualitative	Direct	Teaching career
107	GS	2012	Article	The Journal of Educational Research	Hughes G. D. (2012).	Teacher retention: Teacher characteristics school characteristics organizational characteristics and teacher efficacy.	USA	Quantitative	Indirect	Teaching career
108	GS	2016	Article	FWU Journal of Social Sciences	Husain W. Gulzar A. & Aqeel M. (2016).	The mediating role of depression anxiety and stress between job strain and turnover intentions among male and female teachers.	USA	Quantitative	Indirect	Teaching career
109	WoS CC	2012	Article	Educational Evaluation and Policy Analysis	Ingersoll R. M. & May H. (2012).	The Magnitude Destinations and Determinants of Mathematics and Science Teacher Turnover.	USA	Quantitative	Direct	Teaching career
110	WoS CC	2010	Article	American Educational Research Journal	Ingersoll R. M. & Perda D. (2010).	Is the Supply of Mathematics and Science Teachers Sufficient?	USA	Quantitative	Direct	Teaching career

111	WoS CC	2015	Article	International Migration	Iredale R. R. Voigt-Graf C. & Khoo S. E. (2015).	Trends in International and Internal Teacher Mobility in Three Pacific Island Countries.	Fiji Cook Islands Vanuatu (Pacific Islands)	Quantitative	Indirect	Teaching career
112	WoS CC	2015	Article	Educational Evaluation and Policy Analysis	Jacob R. Goddard R. Kim M. Miller R. & Goddard Y. (2015).	Exploring the Causal Impact of the McREL Balanced Leadership Program on Leadership Principal Efficacy Instructional Climate Educator Turnover and Student Achievement.	USA	Quantitative	Indirect	Teaching career
113	WoS CC	2013	Article	Public Management Review	Johansen M. (2013).	The impact of managerial quality on employee turnover.	USA	Quantitative	Direct	Teaching career
114	WoS CC	2012	Article	Teachers College Record	Johnson S. M. Kraft M. A. & Papay J. P. (2012).	How Context Matters in High-Need Schools: The Effects of Teachers' Working Conditions on Their Professional Satisfaction and Their Students' Achievement.	USA	Quantitative	Indirect	Teaching career
115	WoS CC	2013	Article	Economics of Education Review	Jones M. D. (2013).	Teacher behavior under performance pay incentives.	USA	Quantitative	Direct	Teaching career
116	GS	2016	Article	Global Education Review	Kaden U. Patterson P. P. Healy J. & Adams B. L. (2016).	Stemming the revolving door: teacher retention and attrition in Arctic Alaska schools.	USA	Quantitative	Direct	Teaching career
117	WoS CC	2015	Article	American Educational Research Journal	Kelly S. & Northrop L. (2015).	Early Career Outcomes for the "Best and the Brightest": Selectivity Satisfaction and Attrition in the Beginning Teacher Longitudinal Survey.	USA	Quantitative	Direct	Beginning Teachers
118	WoS CC	2009	Article	Journal of Educational Research	Kukla-Acevedo S. (2009).	Leavers Movers and Stayers: The Role of Workplace Conditions in Teacher Mobility Decisions.	USA	Quantitative	Direct	Teaching career
119	GS	2012	Article	African Journal of Business Management	Kumar D. (2012).	Compensation factors and coping styles: Cross country study on faculty members.	India Pakistan Bangladesh Oman Egypt Indonesia Philippines Malaysia	Quantitative	Indirect	Teaching career
120	GS	2009	Article	Journal of Educational Administration	Kyle Ingle W. (2009).	Teacher quality and attrition in a US school district.	USA	Quantitative	Direct	Teaching career

121	WoS CC	2011	Article	Educational Evaluation and Policy Analysis	Ladd H. F. (2011).	Teachers' Perceptions of Their Working Conditions: How Predictive of Planned and Actual Teacher Movement?	USA	Quantitative	Indirect	Teaching career
122	WoS CC	2017	Article	Teaching and Teacher Education	Lanas M. (2017).	Giving up the lottery ticket: Finnish beginning teacher turnover as a question of discursive boundaries.	Finland	Qualitative	Indirect	Teacher trainees
123	WoS CC	2014	Article	Teaching and Teacher Education	Lavigne A. L. (2014).	Beginning teachers who stay: Beliefs about students.	USA	Quantitative	Indirect	Beginning Teachers
124	WoS CC	2016	Article	Teaching and Teacher Education	Lindqvist P. & Nordanger U. K. (2016).	Already elsewhere - A study of (skilled) teachers' choice to leave teaching.	Sweden	Qualitative	Direct	Teaching career
125	WoS CC	2014	Article	Teaching and Teacher Education	Lindqvist P. Nordanger U. K. & Carlsson R. (2014).	Teacher attrition the first five years - A multifaceted image.	Sweden	Mixed (quantitative and qualitative)	Direct	Beginning Teachers
126	WoS CC	2012	Article	Educational Psychology	Liu S. (2012).	The influences of school climate and teacher compensation on teachers' turnover intention in China.	China	Quantitative	Indirect	Teaching career
127	GS	2008	Article	Teaching and Teacher Education	Liu X. S. & Ramsey J. (2008).	Teachers' job satisfaction: Analyses of the teacher follow-up survey in the United States for 2000-2001.	USA	Quantitative	Indirect	Teaching career
128	GS	2012	Article	Teacher education quarterly	Lloyd M. E. R. & Sullivan A. (2012).	Leaving the profession: The context behind one quality teacher's professional burn out.	USA	Qualitative	Direct	Beginning Teachers
129	GS	2016	Article	Education and Urban Society	LoCascio S. J. Smeaton P. S. & Waters F. H. (2016).	How induction programs affect the decision of alternate route urban teachers to remain teaching.	USA	Mixed (quantitative and qualitative)	Indirect	Beginning Teachers
130	GS	2016	Report/ Technical Report	Regional Educational Laboratory Appalachia	Lochmiller C. R. Sugimoto T. J. & Muller P. A. (2016).	Teacher Retention Mobility and Attrition in Kentucky Public Schools from 2008 to 2012.C387A	USA	Quantitative	Direct	Teaching career
131	WoS CC	2009	Article	Hispania a Journal Devoted to the Teaching of Spanish and Portuguese	Lopez-Gomez C. & Albright J. J. (2009).	Working Conditions of Foreign Language Teachers: Results from a Pilot Survey.	USA	Quantitative	Indirect	Teaching career

132	WoS CC	2014	Article	Physical Education and Sport Pedagogy	Makela K. Hirvensalo M. Laakso L. & Whipp P. R. (2014).	Physical education teachers in motion: an account of attrition and area transfer.	Finland	Quantitative	Direct	Teaching career
133	WoS CC	2015	Article	Journal of Teaching in Physical Education	Makela K. Hirvensalo M. & Whipp P. (2015).	Determinants of PE Teachers Career Intentions.	Finland	Quantitative	Indirect	Teaching career
134	WoS CC	2015	Article	Sa Journal of Human Resource Management	Makhuzeni B. & Barkhuizen E. N. (2015).	The effect of a total rewards strategy on school teachers' retention	South Africa	Qualitative	Indirect	Teaching career
135	WoS CC	2016	Article	English in Australia	Manuel J. & Carter D. (2016).	Sustaining Hope and Possibility: Early-Career English Teachers' Perspectives on Their First Years of Teaching.	Australia	Qualitative	Indirect	Beginning Teachers
136	WoS CC	2008	Article	Teachers College Record	Margolis J. (2008).	What Will Keep Today's Teachers Teaching? Looking for a Hook as a New Career Cycle Emerges.	USA	Qualitative	Indirect	Beginning Teachers
137	GS	2013	Report/ Technical Report	The Research Alliance for New York City Schools	Marinell W. H. & Coca V. M. (2013).	Who Stays and Who Leaves? Findings from a Three-Part Study of Teacher Turnover in NYC Middle Schools.	USA	Quantitative	Direct	Teaching career
138	WoS CC	2017	Article	Public Administration Review	Marvel J. D. (2017).	Not Seeing Eye to Eye on Frontline Work: Manager-Employee Disagreement and Its Effects on Employees.	USA	Quantitative	Indirect	Teaching career
139	GS	2010	Article	International Journal of Construction Education and Research	McCandless D. W. Bruce R. D. & Gebken R. J. (2010).	Retention of Construction Teachers in Secondary Education.	USA	Quantitative	Direct	Beginning Teachers
140	GS	2017	Article	International Journal of STEM Education	McConnell J. R. (2017).	A model for understanding teachers' intentions to remain in STEM education.	USA	Quantitative	Indirect	Teaching career
141	GS	2012	Article	International Journal of Business and Management Tomorrow	Mehta S.	Faculty Attrition: A Challenge for Private Management Institutions.	India	Quantitative	Indirect	Teaching career

142	GS	2012	Article	Journal of Research in Rural Education (Online)	Miller L. C. (2012).	Situating the rural teacher labor market in the broader context: A descriptive analysis of the market dynamics in New York State	USA	Quantitative	Direct	Teaching career
143	WoS CC	2017	Article	Qualitative Sociology	Nelson J. L. (2017).	Pathways to Green(er) Pastures: Reward Bundles Human Capital and Turnover Decisions in a Semi-Profession.	USA	Qualitative	Direct	Teaching career
144	WoS CC	2017	Article	Teachers and Teaching	Newberry M. & Allsop Y. (2017).	Teacher attrition in the USA: the relational elements in a Utah case study.	USA	Qualitative	Direct	Teaching career
145	WoS CC	2015	Article	Economics of Education Review	Ost B. & Schiman J. C. (2015).	Grade-specific experience grade reassignments and teacher turnover. Economics of Education Review	USA	Quantitative	Direct	Teaching career
146	GS	2010	Report/ Technical Report	John F. Kennedy School of Government.	Owens A. (2010).	The Massachusetts teacher workforce: Status and challenges:	USA	Quantitative	Direct	Teaching career
147	WoS CC	2015	Article	Educational Sciences-Theory & Practice	Ozoglu M. (2015).	Mobility-Related Teacher Turnover and the Unequal Distribution of Experienced Teachers in Turkey.	Turkey	Mixed (quantitative and qualitative)	Direct	Teaching career
148	GS	2009	Report/ Technical Report	New Teacher Center	O'Malley G. M.	Teaching Empowering Leading and Learning Maryland Survey Coalition.	USA	Quantitative	Indirect	Teaching career
149	WoS CC	2017	Article	Educational Researcher	Papay J. P. Bacher-Hicks A. Page L. C. & Marinell W. H. (2017).	The Challenge of Teacher Retention in Urban Schools: Evidence of Variation From a Cross-Site Analysis.	USA	Quantitative	Direct	Teaching career
150	WoS CC	2018	Article	Personality and Individual Differences	Perera H. N. Granziera H. & McIlveen P. (2018).	Profiles of teacher personality and relations with teacher self-efficacy work engagement and job satisfaction.	Australia	Quantitative	Indirect	Teaching career
151	WoS CC	2012	Article	Teachers and Teaching	Peters J. & Pearce J. (2012).	Relationships and early career teacher resilience: a role for school principals.	Australia	Qualitative	Indirect	Beginning Teachers
152	WoS CC	2017	Article	Teaching and Teacher Education	Player D. Youngs P. Perrone F. & Grogan E. (2017).	How principal leadership and person-job fit are associated with teacher mobility and attrition.	USA	Quantitative	Direct	Teaching career

153	GS	2011	Article	Australian Journal of Teacher Education	Plunkett M. & Dyson M. (2011).	Becoming a teacher and staying one: Examining the complex ecologies associated with educating and retaining new teachers in rural Australia?	Australia	Quantitative	Indirect	Beginning Teachers
154	GS	2016	Report/ Technical Report	Regional Educational Laboratory Midwest	Podgursky M. Ehlert M. Lindsay J. & Wan Y. (2016).	An Examination of the Movement of Educators within and across Three Midwest Region States. REL 2017-185.	USA	Quantitative	Direct	Teaching career
155	WoS CC	2012	Article	Social Science Research	Price H. E. & Collett J. L. (2012).	The role of exchange and emotion on commitment: A study of teachers.	USA	Quantitative	Indirect	Teaching career
156	WoS CC	2015	Article	Improving Schools	Pyhalto K. Pietarinen J. & Soini T. (2015).	When teaching gets tough Professional community inhibitors of teacher-targeted bullying and turnover intentions.	Finland	Quantitative	Indirect	Teaching career
157	WoS CC	2008	Article	Teachers College Record	Quartz K. H. Thomas A. Anderson L. Masyn K. Lyons K. B. & Olsen B. (2008).	Careers in Motion: A Longitudinal Retention Study of Role Changing Among Early-Career Urban Educators.	USA	Quantitative	Direct	Teaching career
158	WoS CC	2016	Article	American Educational Research Journal	Redding C. & Smith T. M. (2016).	Easy in Easy out: Are Alternatively Certified Teachers Turning Over at Increased Rates?	USA	Quantitative	Direct	Teaching career
159	WoS CC	2017	Article	Teaching Education	Rinke C. R. & Mawhinney L. (2017).	Insights from teacher leavers: push and pull in career development.	USA	Qualitative	Direct	Teaching career
160	WoS CC	2011	Article	Science Education	Ritchie S. M. Tobin K. Hudson P. Roth W. M. & Mergard V. (2011).	Reproducing Successful Rituals in Bad Times: Exploring Emotional Interactions of a New Science Teacher.	Australia	Qualitative	Indirect	Beginning Teachers
161	WoS CC	2018	Article	Educational Policy	Robertson-Kraft C. & Zhang R. S. (2018).	Keeping Great Teachers: A Case Study on the Impact and Implementation of a Pilot Teacher Evaluation System.	USA	Mixed (quantitative and qualitative)	Direct	Teaching career
162	WoS CC	2013	Article	American Educational Research Journal	Ronfeldt M. Loeb S. & Wyckoff J. (2013).	How Teacher Turnover Harms Student Achievement.	USA	Quantitative	Direct	Teaching career

163	GS	2008	Article	American String Teacher	Russell J. (2008).	The Projected Career Plans of String Music Educators: Implications for the Profession.	USA	Quantitative	Indirect	Teaching career
164	WoS CC	2008	Article	Journal of Research in Music Education	Russell J. A. (2008).	A Discriminant Analysis of the Factors Associated With the Career Plans of String Music Educators.	USA	Quantitative	Indirect	Teaching career
165	WoS CC	2012	Article	Bulletin of the Council for Research in Music Education(	Russell J. A. (2012).	The Short and Long-Term Career Plans of Secondary Music Educators: Characteristics of Stayers Movers and Leavers.	USA	Quantitative	Indirect	Teaching career
166	WoS CC	2017	Article	Teaching and Teacher Education	Ryan S. V. von der Embse N. P. Pendergast L. L. Saeki E. Segool N. & Schwing S. (2017).	Leaving the teaching profession: The role of teacher stress and educational accountability policies on turnover intent	USA	Quantitative	Indirect	Teaching career
167	WoS CC	2013	Article	Research in Science Education	Saka Y. Southerland S. A. Kittleson J. & Hutner T. (2013).	Understanding the Induction of a Science Teacher: The Interaction of Identity and Context.	USA	Qualitative	Indirect	Beginning Teachers
168	WoS CC	2011	Article	American Journal of Education	Santoro D. A. (2011).	Good Teaching in Difficult Times: Demoralization in the Pursuit of Good Work.	USA	Qualitative	Indirect	Teaching career
169	WoS CC	2011	Article	Teachers College Record	Santoro D. A. & Morehouse L. (2011).	Teaching's Conscientious Objectors: Principled Leavers of High-Poverty Schools.	USA	Qualitative	Direct	Teaching career
170	GS	2012	Article	National Teacher Education Journal	Sedivy-Benton A. L. & Boden McGill C. J. (2012).	Significant Factors for Teachers' Intentions to Stay or Leave the Profession: Teacher Influence on School Perception of Control and Perceived Support.	USA	Quantitative	Indirect	Teaching career
171	WoS CC	2014	Article	Asia-Pacific Journal of Teacher Education	Shann S. Germantse H. Pittard L. & Cunneen R. (2014).	Community and conversation: tackling beginning teacher doubt and disillusionment.	USA	Qualitative	Indirect	Beginning Teachers
172	WoS CC	2009	Article	Australian Journal of Education	Sharplin E. (2009).	Bringing them in: The experiences of imported and overseas-qualified teachers.	Australia	Qualitative	Direct	Teaching career

173	WoS CC	2014	Article	Educational Research	Sharplin E. D. (2014).	Reconceptualising out-of-field teaching: experiences of rural teachers in Western Australia.	Australia	Qualitative	Direct	Teaching career
174	GS	2018	Article	National Institute Economic Review	Sims S. & Allen R. (2018).	Identifying Schools With High Usage and High Loss of Newly Qualified Teachers.	England	Quantitative	Direct	Beginning Teachers
175	WoS CC	2017	Article	Teachers and Teaching	Smith K. & Ulvik M. (2017).	Leaving teaching: lack of resilience or sign of agency?	Norway	Qualitative	Direct	Beginning Teachers
176	GS	2009	Article	Education Policy Analysis Archives	Sohn K. (2009).	Teacher turnover: An issue of workgroup racial diversity.	USA	Quantitative	Direct	Teaching career
177	WoS CC	2017	Article	Modern Journal of Language Teaching Methods	Soozandehfar S. M. A. & Adeli M. R. (2017).	Factors contributing to Iranian efl teachers' burnout and retention.	Iran	Qualitative	Indirect	Teaching career
178	WoS CC	2016	Article	Educational Evaluation and Policy Analysis	Springer M. G. Swain W. A. & Rodriguez L. A. (2016).	Effective Teacher Retention Bonuses: Evidence From Tennessee.	USA	Quantitative	Direct	Teaching career
179	WoS CC	2016	Article	Journal of Education Finance	Springer M. G. & Taylor L. L. (2016).	Designing Incentives for Public School Teachers: Evidence from a Texas Incentive Pay Program.	USA	Quantitative	Direct	Teaching career
180	WoS CC	2015	Article	Economics of Education Review	Steele J. L. Pepper M. J. Springer M. G. & Lockwood J. (2015).	The distribution and mobility of effective teachers: Evidence from a large urban school district.	USA	Quantitative	Direct	Teaching career
181	WoS CC	2016	Article	Journal of Professional Capital and Community	Struyve C. Daly A. Vandecandelaere M. Meredith C. Hannes K. & De Fraine B. (2016).	More than a mentor The role of social connectedness in early career and experienced teachers' intention to leave.	Belgium	Quantitative	Indirect	Beginning Teachers
182	WoS CC	2014	Article	Teaching and Teacher Education	Struyven K. & Vanthournout G. (2014).	Teachers' exit decisions: An investigation into the reasons why newly qualified teachers fail to enter the teaching profession or why those who do enter do not continue teaching.	Belgium	Quantitative	Direct	Beginning Teachers
183	WoS CC	2012	Article	Pedagogical Studies	Struyven K. Vrancken S. Brepoels K. Engels N. & Lombaerts K. (2012).	Being a teacher? Thanks but no thanks. An investigation into the motives for teachers in Flanders not being willing to start or	Belgium	Quantitative	Direct	Beginning Teachers

						continue teaching five years after graduation.				
184	WoS CC	2012	Article	Economics of Education Review	Stuit D. A. & Smith T. M. (2012).	Explaining the gap in charter and traditional public school teacher turnover rates.	USA	Quantitative	Direct	Teaching career
185	GS	2017	Report/ Technical Report	Regional Educational Laboratory Southwest	Sullivan K. Barkowski E. Lindsay J. Lazarev V. Nguyen T. Newman D. & Lin L. (2017).	Trends in Teacher Mobility in Texas and Associations with Teacher Student and School Characteristics. REL 2018-283.	USA	Quantitative	Direct	Teaching career
186	WoS CC	2017	Article	School Effectiveness and School Improvement	Sun M. Saultz A. & Ye Y. (2017).	Federal policy and the teacher labor market: exploring the effects of NCLB school accountability on teacher turnover.	USA	Quantitative	Direct	Teaching career
187	WoS CC	2012	Article	Canadian Modern Language Review-Revue Canadienne Des Langues Vivantes	Swanson P. (2012).	Second/Foreign Language Teacher Efficacy and its Relationship to Professional Attrition.	USA and Canada	Quantitative	Indirect	Teaching career
188	WoS CC	2014	Article	Hispania-a Journal Devoted to the Teaching of Spanish and Portuguese	Swanson P. (2014).	The Power of Belief: Spanish Teachers' Sense of Efficacy and Student Performance on the National Spanish Examinations.	Spain	Quantitative	Indirect	Teaching career
189	WoS CC	2010	Article	Hispania-a Journal Devoted to the Teaching of Spanish and Portuguese	Swanson P. B. (2010).	Teacher Efficacy and Attrition: Helping Students at Introductory Levels of Language Instruction Appears Critical.	USA	Quantitative	Indirect	Teaching career
190	WoS CC	2009	Article	Journal of Teacher Education	Swars S. L. Meyers B. Mays L. C. & Lack B. (2009).	A Two-Dimensional Model of Teacher Retention and Mobility Classroom Teachers and Their University Partners Take a Closer Look at a Vexing Problem.	USA	Mixed (quantitative and qualitative)	Indirect	Teaching career

191	WoS CC	2010	Article	Teaching and Teacher Education	Tamir E. (2010).	The retention question in context-specific teacher education: Do beginning teachers and their program leaders see teachers' future career eye to eye.	USA	Qualitative	Indirect	Pre-services teachers
192	WoS CC	2011	Article	Teaching and Teacher Education	Tickle B. R. Chang M. D. & Kim S. (2011).	Administrative support and its mediating effect on US public school teachers.	USA	Quantitative	Indirect	Teaching career
193	WoS CC	2015	Article	Cambridge Journal of Education	Tiplic D. Brandmo C. & Elstad E. (2015).	Antecedents of Norwegian beginning teachers' turnover intentions.	Norway	Quantitative	Indirect	Beginning Teachers
194	GS	2016	Article	Journal of School Leadership	Torres A. C.	The Uncertainty of High Expectations: How Principals Influence Relational Trust and Teacher Turnover in No Excuses Charter Schools	USA	Qualitative	Indirect	Teaching career
195	WoS CC	2016	Article	Urban Education	Torres A. C.	Is This Work Sustainable? Teacher Turnover and Perceptions of Workload in Charter Management Organizations.	USA	Quantitative	Direct	Teaching career
196	GS	2016	Article	Journal of School Choice	Torres A. C. (2016).	Teacher efficacy and disciplinary expectations in charter schools: Understanding the link to teachers' career decisions	USA	Qualitative	Direct	Teaching career
197	GS	2012	Article	Journal of Educational Change	Torres A. S. (2012).	"Hello goodbye: Exploring the phenomenon of leaving teaching early.	USA	Mixed (quantitative and qualitative)	Direct	Beginning Teachers
198	WoS CC	2018	Article	Journal of Educational Administration	Torres D. G. (2018).	Distributed leadership and teacher job satisfaction in Singapore.	Singapore	Quantitative	Indirect	Teaching career
199	WoS CC	2017	Article	Teachers and Teaching	Towers E. & Maguire M. (2017).	Leaving or staying in teaching: a 'vignette' of an experienced urban teacher 'leaver' of a London primary school.	England	Qualitative	Direct	Teaching career
200	WoS CC	2017	Article	Research Papers in Education	Trent J (2017).	Discourse agency and teacher attrition: exploring stories to leave by amongst former early career English language teachers in Hong Kong.	Hong Kong	Qualitative	Direct	Beginning Teachers

201	WoS CC	2015	Article	Teachers and Teaching	Tricarico K. M. Jacobs J. & Yendol-Hoppey D. (2015).	Reflection on their first five years of teaching: understanding staying and impact power.	USA	Qualitative	Indirect	Beginning Teachers
202	WoS CC	2010	Article	Educational Psychology	Tsouloupas C. N. Carson R. L. Matthews R. Grawitch M. J. & Barber L. K. (2010).	Exploring the association between teachers' perceived student misbehaviour and emotional exhaustion: the importance of teacher efficacy beliefs and emotion regulation.	USA	Quantitative	Indirect	Teaching career
203	WoS CC	2014	Article	Research on Aging	Van Droogenbroeck F. & Spruyt B. (2014).	To Stop or Not to Stop: An Empirical Assessment of the Determinants of Early Retirement Among Active and Retired Senior Teachers.	Belgium	Quantitative	Direct	Teaching career
204	WoS CC	2016	Article	International Journal of Aging & Human Development	Van Droogenbroeck F. & Spruyt B. (2016).	I Ain't Gonna Make It. Comparing Job Demands-Resources and Attrition Intention Between Senior Teachers and Senior Employees of Six Other Occupational Categories in Flanders.	Belgium	Quantitative	Indirect	Teaching career
205	GS	2015	Article	Journal of Educational Administration	Van Maele D. & Van Houtte M. (2015).	Trust in school: a pathway to inhibit teacher burnout?	Belgium	Quantitative	Indirect	Teaching career
206	WoS CC	2017	Article	Educational Review	Vekeman E. Devos G. Valcke M. & Rosseel Y. (2017).	Do teachers leave the profession or move to another school when they don't fit?	Belgium	Quantitative	Indirect	Teaching career
207	GS	2017	Article	Educational Management	Vekeman E. Devos G. Valcke M. & Rosseel Y. (2017).	Principals' configuration of a bundle of human resource practices. Does it make a difference for the relationship between teachers' fit job satisfaction and intention to move to another school?	Belgium	Mixed (quantitative and qualitative)	Indirect	Teaching career
208	GS	2018	Article	Teaching and Program Development	Watson J. M. (2018).	Job Embeddedness May Hold the Key to the Retention of Novice Talent in Schools. Educational Leadership and Administration	USA	Quantitative	Indirect	Beginning Teachers
209	GS	2016	Article	Education Leadership Review	Watson J. M. & Olson-Buchanan J. (2016).	Using Job Embeddedness to Explain New Teacher Retention.	USA	Quantitative	Indirect	Beginning Teachers

210	WoS CC	2015	Article	Early Childhood Research Quarterly	Wells M. B. (2015).	Predicting preschool teacher retention and turnover in newly hired Head Start teachers across the first half of the school year.	USA	Quantitative	Indirect	Beginning Teachers
211	GS	2017	Article	Urban Education	Whipp J. L. & Geronime L. (2017).	Experiences that predict early career teacher commitment to and retention in high-poverty urban schools.	USA	Quantitative	Direct	Beginning Teachers
212	WoS CC	2012	Article	Kuram Ve Uygulamada Egitim Bilimleri	Yesil Dagli U. (2012).	America's Public School Kindergarten Teachers' Job Turnover and Associated Factors	USA	Quantitative	Direct	Teaching career
213	WoS CC	2017	Article	Teachers and Teaching	Yinon H. & Orland-Barak L. (2017).	Career stories of Israeli teachers who left teaching: a salutogenic view of teacher attrition.	Israel	Qualitative	Direct	Teaching career
